# Unusual CD34 positivity in acute myeloid leukaemia with myelodysplasia‐related changes with megakaryoblastic differentiation

**DOI:** 10.1002/jha2.289

**Published:** 2021-10-01

**Authors:** Daniele Avenoso, Pramila Krishnamurthy, Jonathan Salisbury, Sabia Rashid, Shreyans Gandhi

**Affiliations:** ^1^ Department of Haematological Medicine King's College Hospital NHS Foundation Trust London UK; ^2^ Department of Histopathology, King's College Hospital NHS Foundation Trust London UK; ^3^ Department of Haematology Queen Elizabeth Hospital London UK

## Abstract

Acute megakaryoblastic leukaemia is a rare entity with distinct immunophenotype profile and cytogenetic lesions. Herein, we report a case of acute myeloid leukaemia with myelodysplastic‐related changes with megakaryoblastic differentiation in absence of recurrent genomic lesions such as t(1;22), inv(3) and t(3;3).

One of the peculiarities of this case is the positivity of CD34+ within the abnormal megakaryoblasts; CD61 immunohistochemistry highlights the heavily infiltration of bone marrow from abnormal megakaryoblasts.

A 64‐year‐old male with a known diagnosis of myelodysplastic syndrome/myeloproliferative disorder (MDS/MPN) progressed to blastic phase disease.

Histology of the trephine biopsy section showed large multinucleated cells, which were CD34+ and CD61+ on immunohistochemistry (IHC) in keeping with a diagnosis of secondary acute megakaryoblastic leukaemia (sAML). Cytogenetic studies showed the presence of a complex karyotype:53∼69, including +1,‐5,‐7,+8 and del 17p.


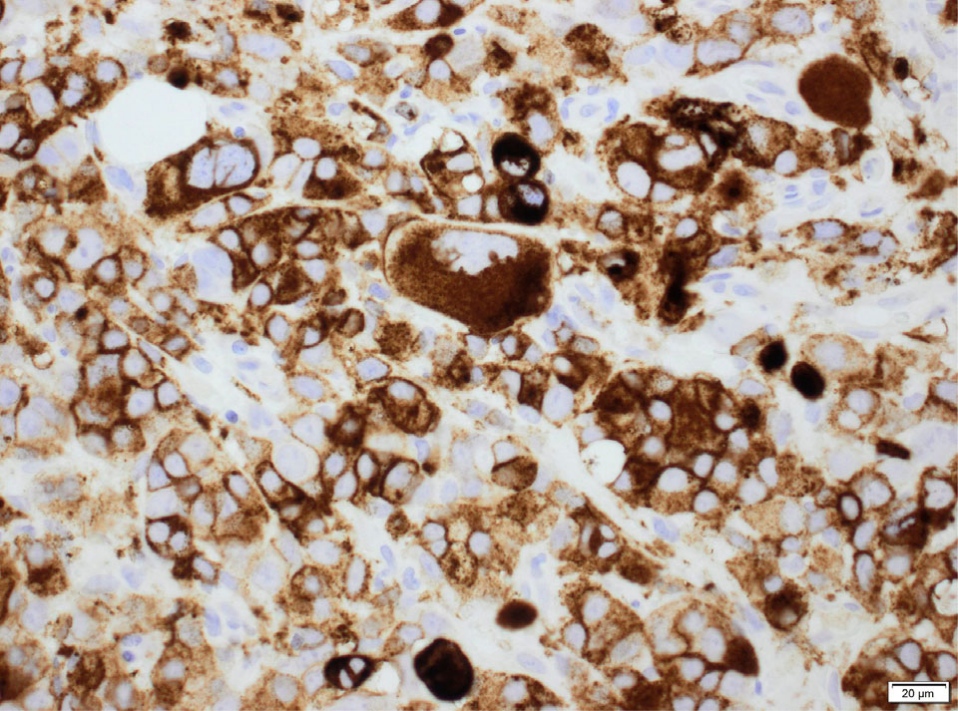


Despite standard induction treatment for high‐risk sAML, the patient never achieved complete remission even if there was a transient reduction of the leukaemic population with disappearance of complex karyotype, suggestive of clonal evolution.


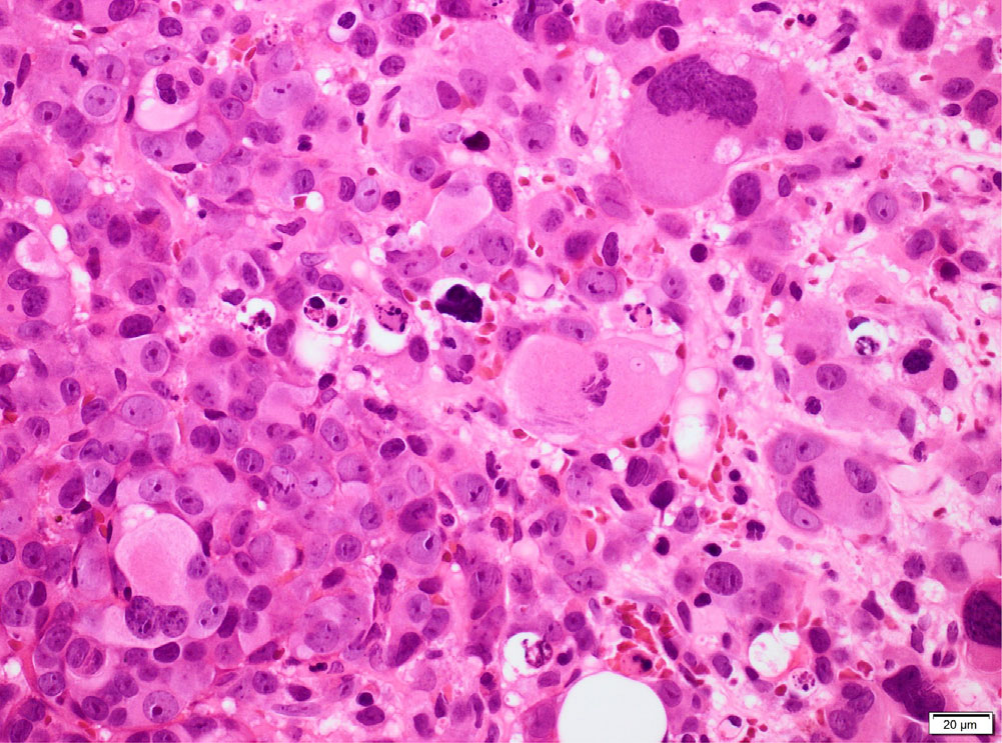


The megakaryocytes/blasts appeared highly dysplastic with abundant cytoplasm and vesicular nuclei and one or more prominent nucleoli. The background stroma appeared fibrotic. Mature erythroid cells were absent. IHC demonstrated megakaryocytes and megakaryoblasts staining strongly for CD34, CD61 but weakly for CD117.

Usually de novo megakaryoblastic leukaemia is CD34 negative and associated with normal karyotype or recurrent genomic lesions such as t(1;22), inv(3) and t(3;3).

The background history of myelodysplastic syndrome/myeloproliferative disorder (MDS/MPN) and the disappearance of the complex karyotype in the persisting blast population suggest the clonal ontogeny in this disease. Furthermore, CD34 expression on the megakaryoblasts may be a consequence of the severe genetic damage secondary to complex karyotype.

## AUTHOR CONTRIBUTIONS

Daniele Avenoso, Pramila Krishnamurthy, Sabia Rashid and Shreyans Gandhi were involved in the care of the patient and wrote the manuscript. Jonathan Salisbury was involved in the diagnosis of the case, took photos and wrote the manuscript.

